# Current Understanding of the Anatomy, Physiology, and Magnetic Resonance Imaging of Neurofluids: Update From the 2022 “ISMRM Imaging Neurofluids Study group” Workshop in Rome

**DOI:** 10.1002/jmri.28759

**Published:** 2023-05-04

**Authors:** Nivedita Agarwal, Laura D. Lewis, Lydiane Hirschler, Leonardo Rivera Rivera, Shinji Naganawa, Swati Rane Levendovszky, Geir Ringstad, Marijan Klarica, Joanna Wardlaw, Costantino Iadecola, Cheryl Hawkes, Roxana Octavia Carare, Jack Wells, Erik N.T.P. Bakker, Vartan Kurtcuoglu, Lynne Bilston, Maiken Nedergaard, Yuki Mori, Marcus Stoodley, Noam Alperin, Mony de Leon, Matthias J.P. van Osch

**Affiliations:** 1 Neuroradiology Unit, Scientific Institute IRCCS E. Medea, Bosisio Parini, Italy; 2 Department of Biomedical Engineering, Boston University, Boston, Massachusetts, USA; 3 Athinoula A. Martinos Center for Biomedical Imaging, Massachusetts General Hospital, Boston, Massachusetts, USA; 4 Department of Electrical Engineering and Computer Science, Massachusetts Institute of Technology, Cambridge, Massachusetts, USA; 5 C.J. Gorter MRI Center, Department of Radiology, Leiden University Medical Center, Leiden, The Netherlands; 6 Wisconsin Alzheimer’s Disease Research Center, University of Wisconsin School of Medicine and Public Health, Madison, Wisconsin, USA; 7 Department of Radiology, Nagoya University Graduate School of Medicine, Nagoya, Japan; 8 Brain Imaging Core, University of Washington, Seattle, Washington D.C., USA; 9 Department of Radiology, Oslo University Hospital Rikshospitalet, Oslo, Norway; 10 Department of Geriatrics and Internal Medicine, Sorlandet Hospital, Arendal, Norway; 11 Department of Pharmacology and Croatian Institute of Brain Research, University of Zagreb School of Medicine, Zagreb, Croatia; 12 Centre for Clinical Brain Sciences and UK Dementia Research Institute Centre, University of Edinburgh, Edinburgh, UK; 13 Biomedical and Life Sciences, Lancaster University, Lancaster, UK; 14 University of Southampton, Southampton, UK; 15 UCL Centre for Advanced Biomedical Imaging, University College of London, London, UK; 16 Biomedical Engineering and Physics, Amsterdam UMC, University of Amsterdam, Amsterdam Cardiovascular Sciences, Amsterdam, The Netherlands; 17 Institute of Physiology, University of Zurich, Zurich, Switzerland; 18 Neuroscience Research Australia and UNSW Medicine, Sydney, Australia; 19 Center for Translational Neuromedicine, University of Rochester Medical Center, Rochester, New York, USA; 20 Center for Translational Neuromedicine, University of Copenhagen, Copenhagen, Denmark; 21 Faculty of Medicine, Health and Human Sciences, Macquarie University, Sydney, Australia; 22 Department of Neurosurgery, Macquarie University Hospital, Sydney, Australia; 23 Department of Radiology and Biomedical Engineering, Miller School of Medicine, University of Miami, Miami, Florida, USA; 24 Weil Cornell Medicine, Department of Radiology, Brain Health Imaging Institute, New York City, New York, USA

## Abstract

Neurofluids is a term introduced to define all fluids in the brain and spine such as blood, cerebrospinal fluid, and interstitial fluid. Neuroscientists in the past millennium have steadily identified the several different fluid environments in the brain and spine that interact in a synchronized harmonious manner to assure a healthy microenvironment required for optimal neuroglial function. Neuroanatomists and biochemists have provided an incredible wealth of evidence revealing the anatomy of perivascular spaces, meninges and glia and their role in drainage of neuronal waste products. Human studies have been limited due to the restricted availability of noninvasive imaging modalities that can provide a high spatiotemporal depiction of the brain neurofluids. Therefore, animal studies have been key in advancing our knowledge of the temporal and spatial dynamics of fluids, for example, by injecting tracers with different molecular weights. Such studies have sparked interest to identify possible disruptions to neurofluids dynamics in human diseases such as small vessel disease, cerebral amyloid angiopathy, and dementia. However, key differences between rodent and human physiology should be considered when extrapolating these findings to understand the human brain. An increasing armamentarium of noninvasive MRI techniques is being built to identify markers of altered drainage pathways. During the three-day workshop organized by the International Society of Magnetic Resonance in Medicine that was held in Rome in September 2022, several of these concepts were discussed by a distinguished international faculty to lay the basis of what is known and where we still lack evidence. We envision that in the next decade, MRI will allow imaging of the physiology of neurofluid dynamics and drainage pathways in the human brain to identify true pathological processes underlying disease and to discover new avenues for early diagnoses and treatments including drug delivery.

## Introduction

The term “Neurofluids” first appeared in the literature in 2019 as an umbrella term to define all fluids in the brain and spine; these being the arterial blood, the venous blood, the cerebrospinal fluid (CSF) and the interstitial fluid (ISF).^1^

The importance of fluids in the body was described in the second century by Galen of Pergamon. He described four fluids, or humors, that sustained life and postulated that disruption of these caused disease. However, it was only in the 13th century, when anatomical dissections were legally allowed, that a more sophisticated macroscopic anatomy of the brain and spine was revealed.^2^ Over the following few centuries and with the development of Galileo’s microscope, neuroanatomists such as Mondino dei Luzzi, Marcello Malpighi, Antoni van Leeuwenhoek, Niels Stensen, Frederic Ruysch, and Domenico Cotugno, described the finer microscopic anatomy. Malpighi suggested that the gray matter (GM) was made up of minute glands and that the white matter (WM) was made up of tubes. Giuseppe Zambeccari hypothesized in the mid-17th century that a liquid separated from the blood entered the brain to allow neural activity to occur. This liquid was called the animal spirit or “sugo de nervi” (nerve juice).^3^ In the mid-18th century Domenico Cotugno identified the CSF that filled the ventricles. In the same period Alexander Monro and George Kellie proposed the well-known doctrine that the fixed volume of the skull dictates equal in and outflow of blood, however they both completely ignored the contribution of CSF. Neurosurgeons such as Harvey Cushing and neurologists such as Giulio Bizzozero including anatomists such as Camillo Golgi, were subsequently essential in defining the interrelation between blood (arterial and venous) and CSF flow. Helen Cserr in the mid-20th century pioneered research on the ISF describing its dynamics, thereby laying the groundwork for modern work on neurofluids.^4^

Since the development of the first clinical scanners in early 1980s, several Magnetic Resonance Imaging (MRI) based techniques have been developed to image neurofluids (Table 1). These include methods using exogenous (eg, gadolinium-based imaging techniques, and GBCA) and endogenous (eg, arterial spin labeling methods) tracers. The study of CSF flow dynamics is largely based on phase-contrast techniques including the more novel 4D flow methods. More recently, diffusion tensor imaging (DTI), functional MRI (fMRI) and other techniques are applied to evaluate microstructure and neurovascular oscillations and interactivity of different fluid compartments, leading to clear increase in academic output.

**FIGURE 1:**
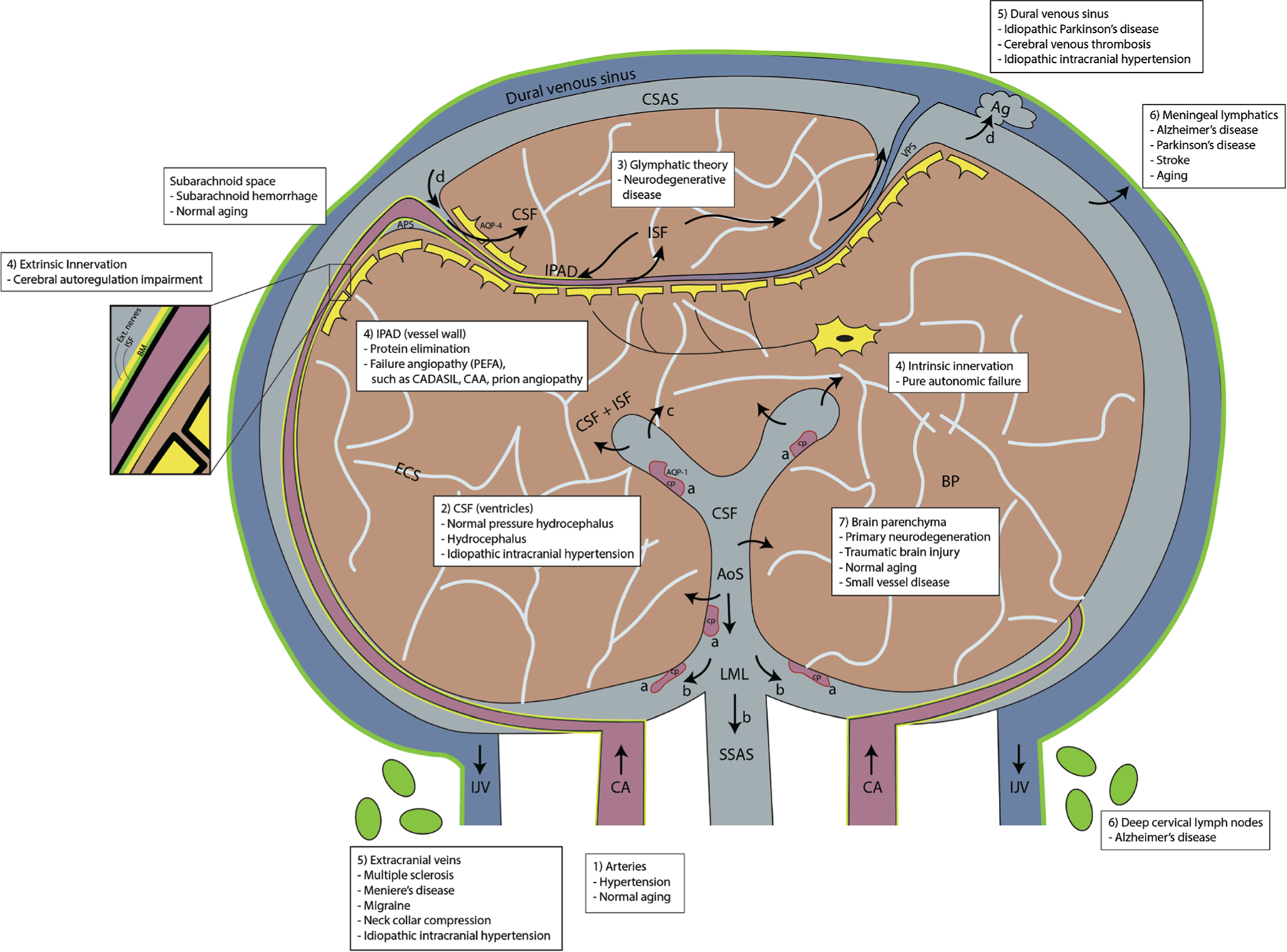
Schematic drawing on a coronal section of the brain illustrating the interaction between neurofluid compartments and BP with possible neurological diseases (white text inserts) that can be a result of their derangement: Leptomeningeal arteries and penetrating arterioles branch out from the carotid arteries (CA) and form a dense network on the surface of the pia in the cerebral subarachnoid space (CSAS). The CSF is (**a**) produced from the CP, flows (**b**) caudally into the aqueduct of Sylvius (AoS) toward the CSAS via the foramen of Luschka and Magendie (LML) and into the spinal subarachnoid space (SSAS), (**c**) may exit via the ventricular ependymal wall toward the BP and also (**d**) CSF drains into the dural venous sinus through Ag. (**e**) CSF can recirculate into the BP via arterial perivascular space (APS) and into the BP through AQP-4 water channels. Bulk flow and/or diffusion processes move ISF/CSF from the BP toward the venous perivascular space (VPS), transporting metabolic waste out toward the CSAS and into the dural venous sinus. IPAD pathway is the flow of ISF in the opposite direction to arterial flow along the BM of arteries (zoomed box) and leaves the intracranial cavity along the walls of the CA into the deep cervical lymph nodes. Both extrinsic and intrinsic innervations line the arterial and capillary walls respectively, and are determinants of cerebral autoregulation. The venous system is represented on the surface of the CSAS. Cortical veins drain into the superficial venous sinus system (dural venous sinus). Venous blood exits out of the intracranial cavity toward the heart via internal jugular veins (IJV) and vertebral veins (not shown). Meningeal lymphatics are channels that line the dural venous sinus, the cranial nerve sheaths, the cribriform plate and also the basal venous plexi, draining CSF via perineural sheaths. BP is made up of 80%–85% of cells and ISF. CADASIL = cerebral autosomal dominant arterial with subcortical infarcts and leukoencephalopathy. CAA = cerebral amyloid angiopathy. (adapted from Agarwal et al^1^).

Within the International Society of Magnetic Resonance in Medicine, a new study group, “Imaging Neurofluids,” was created in 2019, drawing interest from many MR scientists worldwide. The first workshop was held in September 2022 in an endeavor to unite key scientists and clinicians and systematically delineate what is known and what is still to be uncovered. The outcome of this workshop is reflected in this review article. It is no doubt that a better understanding of the dynamics of neurofluids has the potential to greatly enhance our understanding of the physiological pathways of brain clearance, their role in neurological diseases, and the development of new avenues for diagnostics and treatment (Fig. 1).

## Basics of Neurofluids

### Cerebral Hemodynamics

The brain is critically dependent on a well-regulated blood supply matched to the continuously changing metabolic requirements of neurons and glia.^5^ The structural and functional characteristics of the cerebral vessels are designed to assure that it receives sufficient blood supply and that the homeostasis of its microenvironment is preserved. Structurally, the major cerebral vessels merge at the base of the brain in the circle of Willis, which permits compensatory flow through alternate routes in case of arterial obstruction, whereas the pial microcirculation on the brain surface forms a dense anastomotic network that allows blood to reach the depths of the brain through more than one route.^6^ Functionally, the neurovasculature: (a) prevents potentially deleterious reductions in blood flow during drops in blood pressure within a certain pressure range,^7^ (b) matches the delivery of oxygen and glucose with the energy requirements of the active brain, such that increases in neural activity lead to corresponding increases in blood flow to the active regions,^5^ (c) contributes to clearance of metabolic waste generated by the brain,^8^ (d) regulates the bidirectional molecular exchange between blood and brain through transcytosis and molecular transporters,^9^ (e) is involved in the trafficking of immune cells in and out of the brain,^10^ and (f) generates critical growth factors to maintain the vitality of neurons and glia.^11^ In brain diseases, these critical features of the neurovasculature are disrupted. For example, cerebrovascular senescence, vascular risk factors (eg, hypertension, hyperlipidemia, and diabetes) and atherosclerosis cause macrovascular and microvascular occlusions that lead to acute or chronic blood flow reductions causing ischemic strokes or vascular cognitive impairment, respectively.^12^ Alterations in the regulation of the neurovasculature also occurs in neurodegenerative diseases, such as Alzheimer’s disease (AD), frontotemporal dementia, and other proteinopathies (amyloid-β (Aβ), tau, α-synuclein, TDP43, etc.).^5^ In these conditions, alterations in cerebral perfusion, immune dysregulation, blood–brain barrier disruption, trophic failure, and alterations of clearance pathways are often observed and may contribute to brain dysfunction and cognitive impairment.^5,8,13^ Neurovascular health is, therefore, vitally important for the maintenance of brain health. Measures to preserve and promote the well-being of cerebral blood vessels remain a necessary component for prevention strategies of vascular cognitive impairment.

### Perivascular Spaces

**FIGURE 2:**
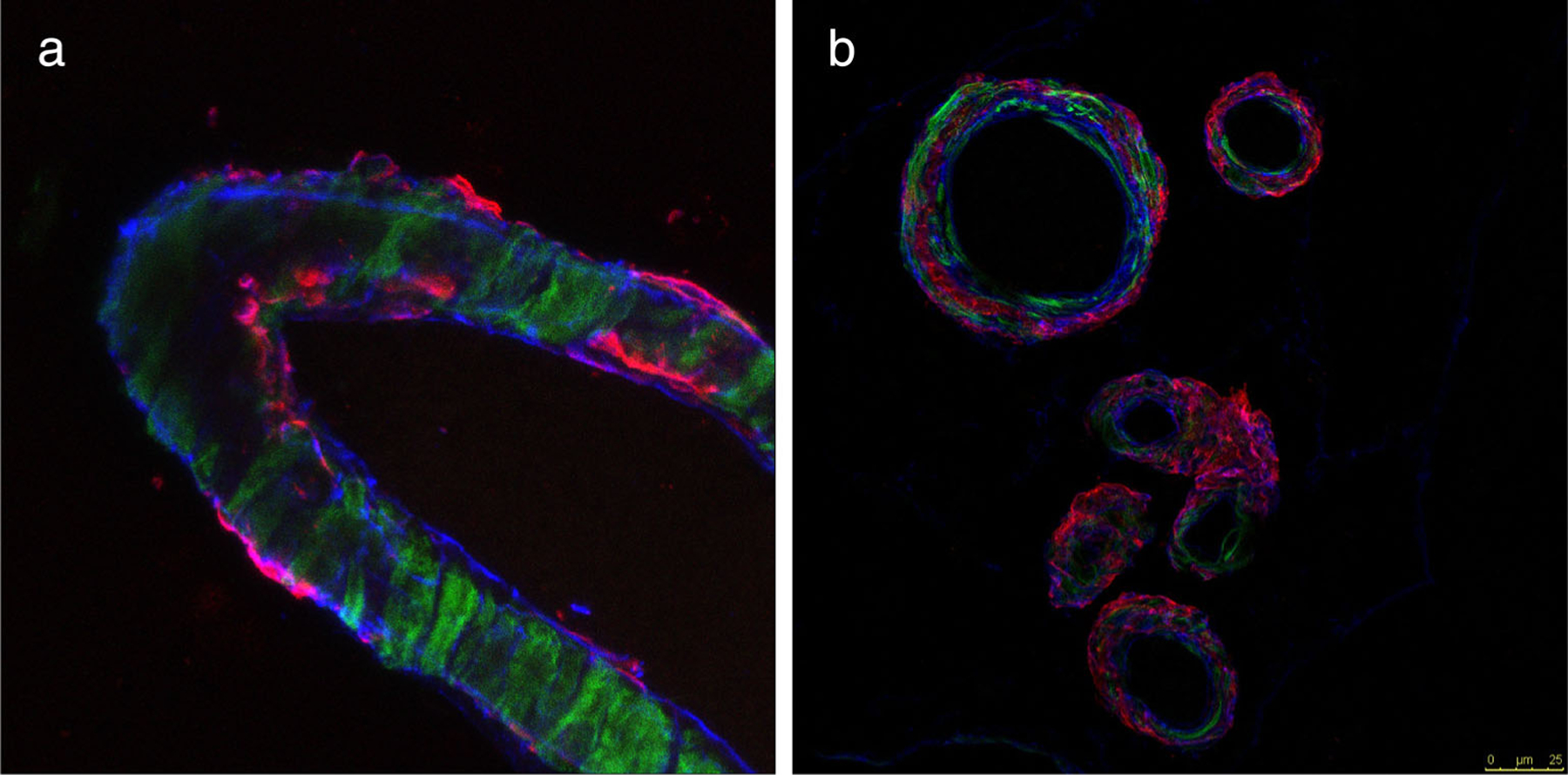
An example of Aβ accumulation within the perivascular drainage pathways (IPAD). (**a**) Histological sample showing human CAA. Aβ (red) is deposited in BMs (collagen IV is in blue) in the walls of a leptomeningeal-artery. (**b**) Cross-sectional sections of leptomeningeal arteries show the smooth muscle. Actin in green and colocalized Aβ in red. (unpublished data).

In vivo experiments using high-resolution two-photon imaging show that the periarterial spaces are large tunnels that allow fast unidirectional forward flow of microspheres in rodents. The velocity of microspheres in perivascular spaces (PVS) surrounding pial arterioles is on the order of ~10–20 μm/sec in mice.^14^ Kinetic data cannot be obtained in the PVS surrounding penetrating arterioles but is likely in the same range as they are filled within minutes with fluorescent dextran tracers (4–100 kDa).^15^ In histological sections, PVS appears as a space between the outer aspect of an artery wall and the brain parenchyma (BP) that can be expanded experimentally by the intracerebral injection of Indian ink particles or fluorescent polystyrene beads.^16,17^ As arteries enter the surface of the cerebral cortex, the pia mater is reflected from the surface of the brain onto the vessels in the subarachnoid space (SAS), thus separating it from the cerebral cortex.^18^ Histological studies are unable to visualise PVS around cortical arteries in the normal brain, as layers of smooth muscle, basement membrane (BM), and one layer of leptomeninges, are all compressed together upon fixation of tissues.^18^ In the WM and in the basal ganglia, however, there are two layers of leptomeninges surrounding the artery with a PVS in between the layers^19^ (Fig. 2). These histological studies have not been confirmed by in vivo studies. A recent study documented that the separation between the PVS by pia mater has no functional significance: CSF tracers enter deep into the brain along the periarterial spaces independent of the degree of pial matter coverage. Using normal adult canine brain tissue it was demonstrated that in the WM, arteries possess two layers of leptomeninges, with a potential space between them.^20^ Branches of the leptomeningeal arteries on the surface of the brain pass through the cerebral cortex, often without branching, to supply the underlying WM.^21^ It is important to note that fluid filled spaces largely disappear after death, since loss of the membrane potential upon energy failure gives rise to uncontrollable cellular swelling. One of the best examples of this is that the extracellular spaces that in vivo contributes to ~20% of the total tissue volume are completely lost in histological sections.^22^ Observation in histological sections should therefore be used with caution when developing models of brain fluid transport. In fact, tracers injected into CSF will, in highly unphysiological ways, be moved around due to the rapid changes in intracranial pressure (ICP) upon death.^23^ That publication displayed a movie of CSF tracer displacement during cardiac perfusion with 4% paraformaldehyde from the PVS to inside the vessel wall (Video S1).

**FIGURE 3:**
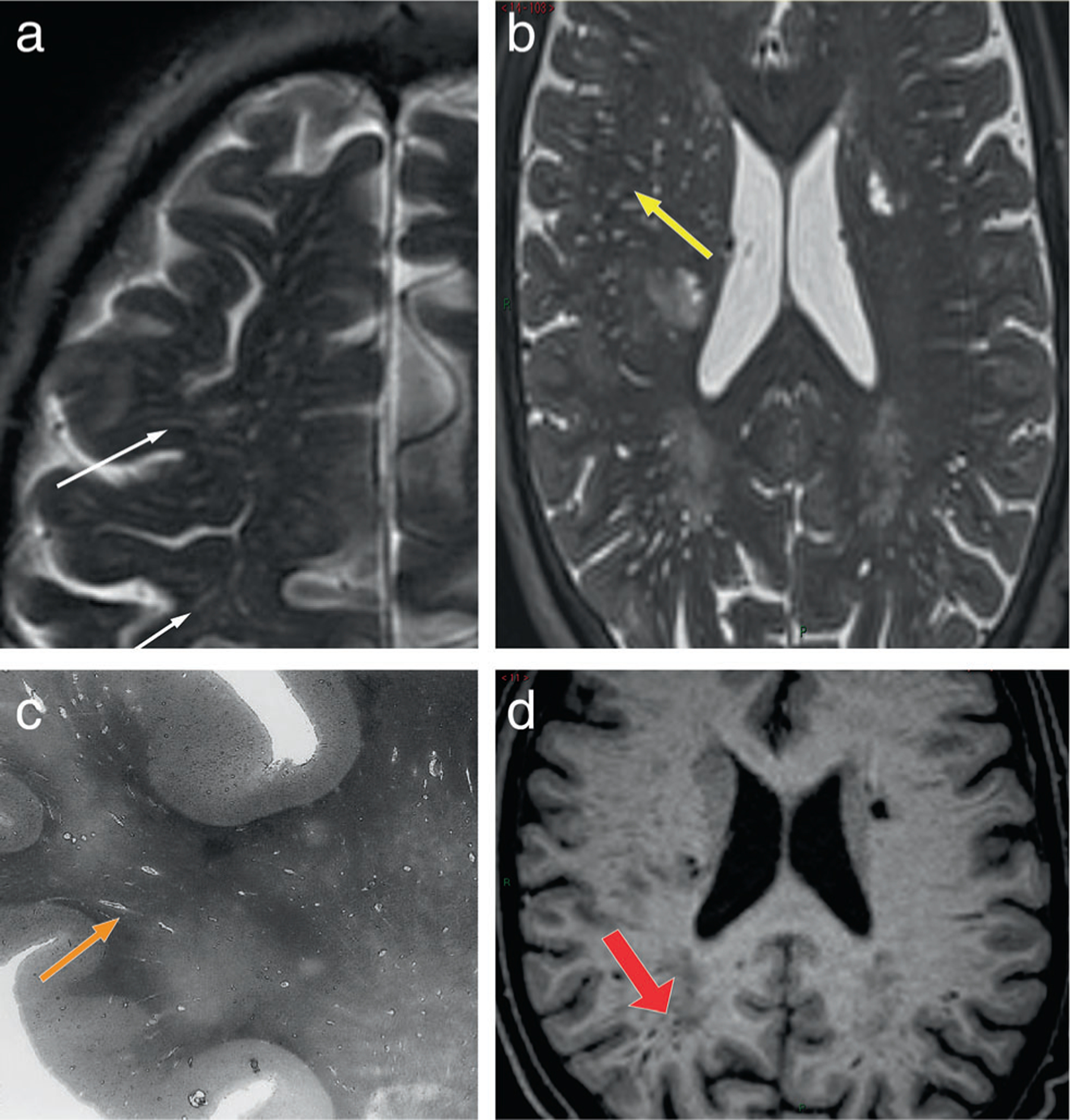
The appearance of PVS on conventional MR images. (**a**) axial T2-weighted image at the level of the centrum semiovale, show multiple thin, linear structures presenting a hyperintense signal similar to CSF signal (white thin arrows); (**b**) axial T2-weighted image at the level of the lateral ventricles shows some dotted structures presenting CSF-like signal (yellow arrow). The morphology of PVS (linear vs. dot) depends on whether the vessel is parallel or perpendicular to the plane of the image. (**c**) a histologic slide showing multiple fluid filled linear PVS (orange arrow). (**d**) T1-w axial image shows linear PVS with a hypointense CSF-like signal (red thick arrow).

PVSs are visible on MRI as small lines or dots of CSF signal <3 mm diameter in the subcortical gray and WM orientated in line with perforating arterioles^24^ (Fig. 3). They are visible when there is some fluid surrounding the arteriole, usually of CSF signal, and are most clearly seen in the basal ganglia and WM of the centrum semiovale. T2-weighted imaging is the most sensitive sequence for visualizing PVS; T1-weighted sequences are less sensitive therefore typically identifying fewer and only larger PVS.^25,26^ Although there are spaces around the venules in the brain, these are generally not visible on MRI.^27^ PVS visibility on MRI increases with age, and the appearance of PVS is highly heritable.^28^ However, PVS visibility depends critically on sequence parameters and field strength. Therefore, more PVS will become MR-visible when using sensitive sequences.^24^ PVS can be quantified using visual scores^29^ or computational methods,^25^ the latter enabling the assessment of PVS number, total and individual PVS volumes, PVS width, length, clustering, and orientation. Such quantitative measures allow to track the relation of PVS-characteristics to development of tissue damage, for example, white matter hyperintensity (WMH) formation.^30^ The clinical diagnostic value of PVS quantification is currently an active area of neurofluids research. Fluid accumulation in enlarged PVS (EPVS), seen on MRI might originate from the accumulation of Aβ in the penetrating artery wall as seen in cerebral amyloid angiopathy (CAA), but may represent an indirect marker of impaired clearance of waste proteins, perivascular inflammation, and overall dysfunction of the blood–brain barrier.^31,32^

### ISF Composition

Among the fluids of the brain, ISF is arguably in closest communication with neurons and glia. Although the exact source of ISF is unknown, it is thought to originate from vascular, CSF, and cellular sources. ISF is water-based and comprises a range of ions, proteins, neurotransmitters, and other signaling molecules.^33^ In addition, ISF contains brain metabolites and other waste products that are generated as a result of the high metabolic turnover of the CNS. The composition and concentration of these products is influenced by numerous factors, such as neuronal activity, circadian rhythm, biological sex and age.^34–36^ Among these, proteins such as Aβ, tau, α-synuclein, and huntingtin have gained particular attention because of their association with neurodegenerative conditions such as AD, Parkinson’s (PD) and Huntington’s disease (HD), respectively. Aβpeptides are secreted into the ISF following the proteolytic cleavage of the amyloid precursor protein. Aβis prone to aggregate into neurotoxic fibrils that comprise extracellular senile plaques that are characteristic of AD, and which can attract binding of soluble Aβ that is present in the ISF.^37^ Thus, the density and biochemical composition of senile plaques can influence the concentration of Aβ within the ISF. Unlike Aβ, tau, α-synuclein, and huntingtin proteins aggregate principally within neurons and glia.^38,39^ However, in vitro and in vivo evidence suggest that pathogenic aggregates of these proteins are actively secreted into the ISF and this secretion may contribute to the intracellular transmission and spread of pathology across brain regions in AD, PD, and HD.^33,40^ The degree to which communication between the ISF, CSF, and the activity of mural cells contributes to the concentration and spread of protein aggregates throughout disease progression is an area of active research in neurofluids.

### CSF Dynamics

The classical understanding of CSF physiology is that the choroid plexi (CP), cisternal spaces, and the arachnoid granulations (Ags) in the dural sinuses are the key anatomical structures that determine constant formation, unidirectional flow and passive absorption of CSF predominantly into the dural venous sinuses.^41,42^ However, after injecting tracers in the ventricles, significant amounts enter the adjacent CNS tissue and disperse into all compartments of the CSF system driven by CSF pulsations. Tracer dispersion can also occur in the opposite direction, i.e., from the SAS toward the lateral ventricles, which supports alternative models of CSF production and absorption beyond the traditional view of “ventriculo-cisternal perfusion.”^43^ This movement of CSF from the ventricles into the BP is in accordance with the metabolite waste clearance concept.^43^ Also, the conventional understanding that Ags are the prime sites of CSF absorption must be revised in the light of new findings that suggest that their number, size and anatomical distribution evolves over the entire human life-course.^44^ Experimental, clinical, and radiological findings point out that the classical hypothesis of CSF physiology is not able to explain numerous phenomena and cases, showing the necessity for renewal of this concept. For example, ASL MRI with a long echo time has recently identified water exchange between subarachnoid CSF and dural arteries.^45^

The pulsatile flow of CSF is likely a key contributor to clearance mechanisms. With every heartbeat, small amounts of CSF move back and forth between the two main compartments of the cranio–spinal (CS) system, i.e., the cranium and the spinal canal. After two centuries of the “discovery of the CSF” by Magendie in 1825, the critical role of these CS CSF-oscillations in maintaining brain health is recognized. These oscillations can facilitate the circulation of the blood through the cerebral vasculature, primarily in the supine posture, when the compliance of the cranium is reduced.^46^ Although CSF oscillations were not part of the original Monro-Kellie doctrine, such oscillations during systole and diastole remain fundamental to a maintain a constant ICP. Phase contrast (PC)-MRI studies have clearly shown that during systole the cranial CSF volume is reduced to accommodate the inflow of arterial blood and vice versa. CSF-oscillations in the CS system are also likely to facilitate the removal of metabolic waste products from the brain which largely occurs during sleep.^47^ In fact, the CS stroke volume (SV) is over 2-fold larger in the supine compared to the upright posture.^46^ The mechanical properties of the tissues of the cranium and spinal canal compartments play a significant role in the regulation of ICP but also in the dynamics of the CS CSF oscillations. The dura mater in the cranium is more tightly restricted by the cranial vault compared with the dura mater in the spinal canal, especially in the lumbar region.^48^ This enables the CSF pulse pressure to propagate further down the spinal canal. It is important to recognize that both the cranium and the spinal systems participate in the effective oscillation of CSF.

## Neurofluids Physiology: Differences Between Rodents and Humans

There are important differences in the anatomy and physiology of rodents and humans that must be considered when extending results from mouse models to humans. At a macroscopic level, the mouse brain is ~10 mm wide, as opposed to the human brain, which is ~140 mm wide.^49^ The human cortex is characterized by folds, i.e., sulci and gyri, as opposed to the lissencephalic mouse cortex. The movement of ISF occurs over large and convoluted spatial geometries in humans. Studies in mice show that this flow occurs preferentially along major WM pathways, which are abundant in humans and sparse in mice.^15^ Although experimental evidence is limited, mass transport models support that fluid transport in humans may be described by advection and diffusion rather than diffusion alone, which may be sufficient for the mice brain.^50,51^ At a microscopic level, astroglial aquaporin 4 (AQP4) water channels localized in perivascular astrocytic endfeet support perivascular fluid and solute exchange. Interestingly, the ratio of AQP4 channels in endothelium to that in the parenchyma is 7:1 in mice but only 2:1 in humans indicating that the astroglia water transport and the parenchyma may play a larger role in mediating fluid transport across the larger human brain.^52^ Fluid transport in mouse models is more active during sleep and increasing evidence suggests this may also be the case in humans. However, mice are nocturnal and experience a highly fragmented sleep structure with rapid eye movement (REM) sleep every 10–15 minutes while humans are diurnal and experience REM sleep approximately every 90–120 minutes.^53^ Whether mice have distinct stages within NREM is unclear; humans have clear NREM sleep stages that can be identified using EEG. Homeostatic drive to sleep increases with age in mice, but decreases with age in humans.^54^ These and other key differences including arterial and venous arborization, meningeal lymphatics, CSF volume, and turnover rates between mice and human models need to be well-characterized before animal observations can be translated to human conditions.

## Models of CSF-Mediated Brain Waste Drainage Pathways

### Traditional Models

**FIGURE 4:**
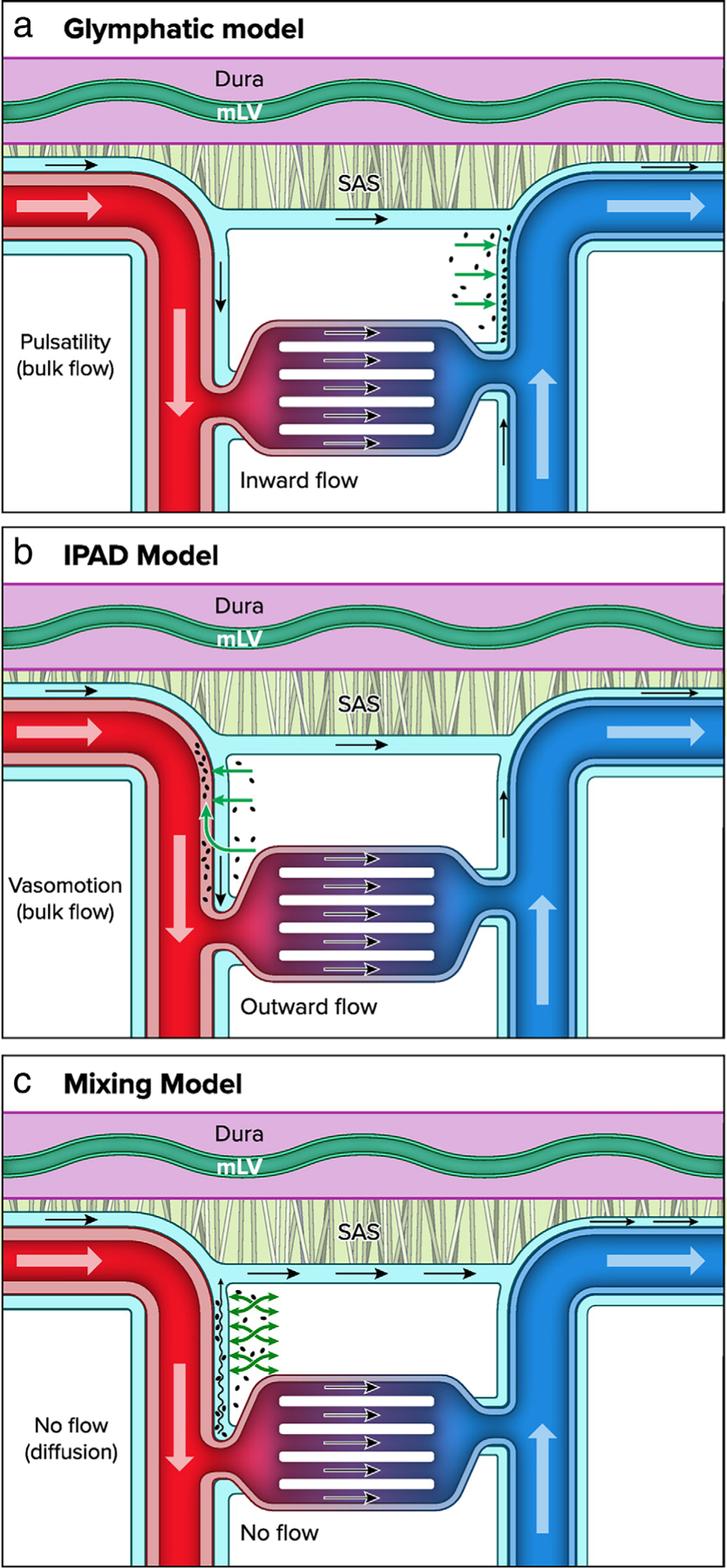
Brain waste clearance pathways: (**a**) glymphatic model describes the flow of ISF and waste products from the paravascular arterial space toward the paravenous space; (**b**) the IPAD suggests that ISF and waste products are cleared via the periarterial space, countercurrent to the main arterial blood flow; (**c**) the mixing model suggests that there is no flow along the penetrating blood vessels, rather ISF and waste products undergoes “mixing” from physiological processes such as arterial pulsatility (adapted from Zhao et al^8^).

Since 2012, the term “glymphatic” (glial-lymphatic) has de facto become an umbrella term used to describe brain clearance via peri/paravascular pathways. It was proposed in 2012, using in vivo two-photon microscopy and dynamic contrast-enhanced magnetic resonance imaging (DCE-MRI)^55,56^ (Fig. 4). The glymphatic system was identified as a macroscopic brain waste clearance network that uses a unique complex of perivascular channels formed by astroglial cells to remove metabolic waste from the interstitial space of the BP. This system entails the inflow of CSF along PVS surrounding arteries followed by AQP4 mediated exchange of CSF into the tissue, where the ISF “flushes” the brain tissue and takes up soluble waste products that subsequently egress via perivenous spaces. In the important follow-up publication, it was shown that this brain-wide astrocyte-mediated transport of CSF and ISF is primarily enhanced during NREM sleep when slow-wave EEG activity is high.^57^ In addition to eliminating waste, the glymphatic system may help circulate essential nutrients and neurotransmitters, and might also act as a pathway for drug delivery.^56^ This suggests that major acute and chronic brain diseases may be better treated by harnessing the glymphatic system. Multiple additional CSF egress drainage pathways have been proposed, including along the cranial nerves and lymphatic vessels.^58–60^

As an alternative, or in addition to the glymphatic model, the intramural periarterial drainage pathway (IPAD) model has been proposed. IPAD lies between the layers of the vascular smooth muscle cells and the vascular BMs^16^ (Fig. 2). This model proposes that the ISF draining toxins such as Aβ enter the IPAD pathway and move intramurally in the opposite direction to arterial blood flow toward the cervical lymph nodes. Fluids and waste products propagate out of the brain centrifugally, which would primarily be supported by the intrinsic contractile activity of the smooth muscle cells, also known as vasomotion.^61,62^

### Beyond Traditional Models: The Mixing Hypothesis

There is strong evidence for bulk flow of CSF at the brain surface.^23,63^ However, whether flow continues along PVS of penetrating vessels is not clear. Flow at the level of the SAS does not necessarily imply continuation of flow along penetrating vessels, conservation of mass may be preserved through multiple parallel exit routes of CSF, including dural lymph vessels, along olfactory nerves through the cribriform plate, and along spinal nerves. These pathways may bypass brain tissue. Recent work showing electron microscopic details of mouse brain tissue shows pial membranes covering the entry sites of penetrating arteries into the brain.^63^ These membranes are expected to prohibit the entry of cells and micrometer sized particles, but perhaps do not restrict flow of water and small solutes. This anatomical barrier however limits approaches to determine flow characteristics based on particle tracking, usually performed via in vivo imaging techniques in rodents.

While actual data on perivascular flow patterns along penetrating vessels are currently lacking, modeling work suggested that bulk flow is not necessary to enhance exchange of solutes between the CSF and the brain ISF.^64^ Mixing of fluid may also provide an efficient mechanism of solute exchange, which allows both waste clearance from the brain, as well as entry of nutrients and other soluble factors from the CSF to the interstitium along the same pathway.^8,65^ This pathway is most likely represented by spaces along penetrating arteries, as PVS along veins appear to be limited or very small.^27^ In this view, the continuous supply of “fresh” CSF at the level of the SAS is necessary to provide a concentration gradient for waste clearance. The PVS along penetrating vessels then provide low resistance pathways where oscillations induce mixing, thereby greatly enhancing the exchange between CSF and ISF.

The oscillations that have been found thus far to enhance tracer dispersion in rodents include vascular pulsations induced by the heartbeat,^66^ and vasomotion.^62^ Respiration has also been suggested as a driving force, especially in humans.^67^ These potential driving forces of CSF–ISF exchange have been implicated in both the glymphatic^15^ as well as the IPAD^68^ theory. These concepts, however, differ in their anatomical delineation of the drainage routes and direction of flow. Differences in methodology, including tracer injection in CSF versus tracer injection into the parenchyma may play a role herein. Thus, solute dispersion may be dependent on the pressure gradient induced by the infusion itself, or the steep concentration gradient that results from local injection. Both methodological limitations may easily obfuscate the physiological routes of solute movement. Thus, techniques that are minimally invasive, which detach tracer infusion from analysis of solute movement or do not rely on tracer infusion, are currently needed, including the need to research CSF–ISF driving forces directly in humans. Such technical advances may provide definitive answers on the question whether bulk flow or mixing is present in PVS along penetrating arteries (Fig. 4).

## MR Imaging Techniques

### Noncontrast Enhanced Techniques

#### DIFFUSION TENSOR IMAGING.

Intravoxel incoherent motion (IVIM) based diffusion MRI and DTI analysis along the perivascular space (DTI-ALPS) have been employed to study the movement of water molecules within the interstitium as an indirect approach to the study of ISF dynamics.^69,70^ IVIM-based measurements of ISF rely on acquiring data over a broad range of *b*-values with more emphasis on intermediate *b*-values, since ISF is thought to exhibit diffusion values in between the pseudo-diffusion values caused by perfusion within a randomly oriented microvascular bed and tissue diffusion. DTI-ALPS relies on the specific anatomical features of PVS at the level of the lateral ventricle, where the medullary veins are running perpendicular to the main fiber tracks. The main advantage of DTI-ALPS is that it uses traditional DTI sequences and therefore allows retrospective analysis. However, to what extent the DTI-ALPS index is really a marker of glymphatic function is still a subject of active debate.^71^ The main criticisms of this technique include that the signal is not specifically sensitive to CSF, lacks sufficient proof that the measured signal is limited to PVSs, and that visible PVSs adjacent to the lateral ventricle are not perivenular.^27^ However, by combining motion-sensitizing gradients with long echo-time readout, the MR signal can be limited to CSF around major and penetrating arteries, allowing us to measure the mobility of CSF in PVSs.^72^ Another approach for employing diffusion techniques for brain clearance imaging relies on multi-*b*-value acquisitions and splitting of the signal into three compartments: a perfusion and diffusion component, and a third, intermediate component that might reflect CSF or ISF.^73^

#### PC MEASURES OF CSF FLOW.

**FIGURE 5:**
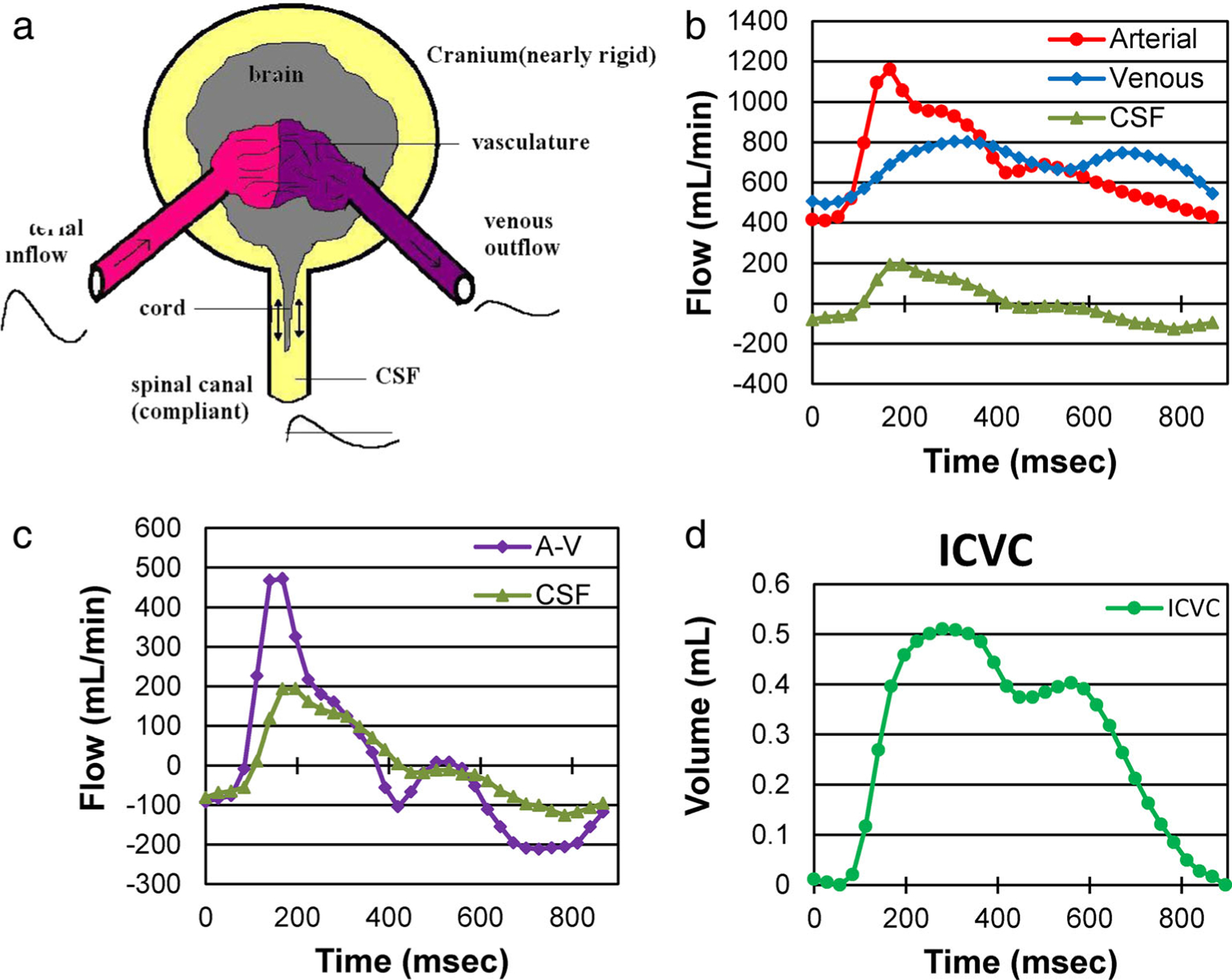
(**a**) A simplified model of the CS system. (**b**) Volumetric flow rate waveforms of the arterial inflow, venous outflow, and the CS CSF flow. (**c**) The cs CSF flow waveform plotted with respect to the arterial minus venous (A-V) flow waveform. The fact that these two waveforms are not identical implies that the ICV is not constant and thus, the cranium has compliance. The CSF waveform follows the pattern of the net transcranial blood flow suggesting that the A-V flow drives the CS CSF pulsation. (**d**) The intracranial volume change during the cardiac cycle obtained by time integration of the next transcranial flow.^48^

The noninvasive quantification of neurofluids dynamics is challenging for several reasons; particularly, the dynamic range of the fluid velocities and the size of the compartments. Quantitative CSF flow measures are typically obtained by PC-MRI.^48^ Some salient observations from PC MRI studies are as follows (Fig. 5). The CS CSF flow pulsatility is driven by a temporary difference in the intracranial arterial inflow and venous outflow during the cardiac cycle and is further modulated by the intracranial compliance, respiration and intrathoracic pressure.^74^ The larger the intracranial compliance the less CSF volume will leave the cranium during systole. Venous outflow also occurs during systole and seems to be the primary source of CSF oscillations. Venous outflow is also influenced by breathing. Breathing is associated with changes in the intrathoracic pressure that directly influences the pressure in the right atrium and also in the epidural veins. During inspiration, the intrathoracic pressure drops creating an inverse pressure gradient between the cranium and the thorax, facilitating venous flow. During expiration, venous return to the heart is blocked by an increase in the Intrathoracic pressure. Changes in epidural vein volume also drive spinal CSF flow.^75^ The in-depth study of the origin and driving force of the CS CSF oscillations has led to the development of MR based quantification of intracranial compliance and pressure as illustrated in Fig. 5.^48^

#### ARTERIAL SPIN LABELING FOR MEASURING WATER TRANSPORT ACROSS BLOOD–CSF BARRIER.

**FIGURE 6:**
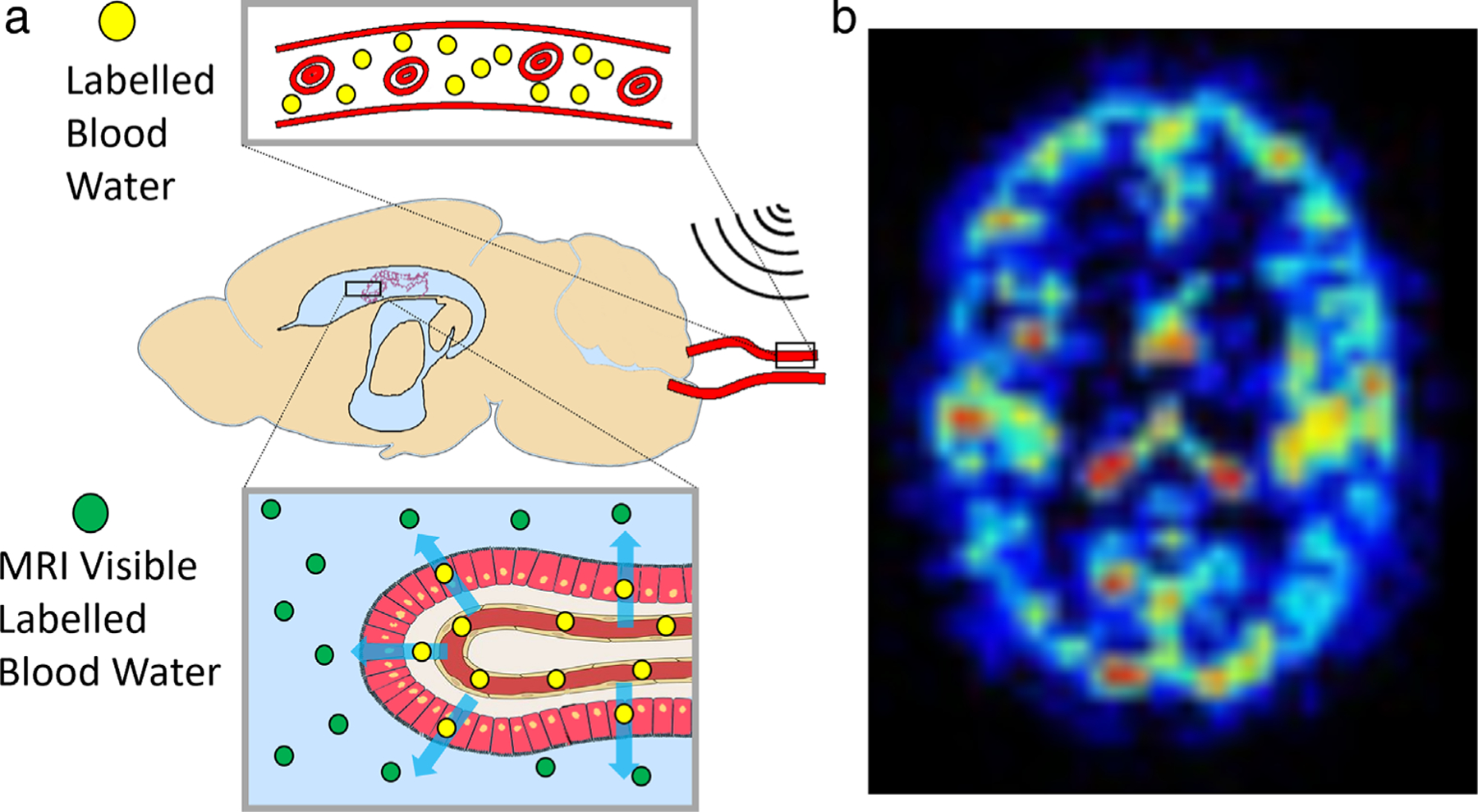
(**a**) Schematic representation of concept proposed in Evans et al,^78^ where the MRI signal is measured from labeled arterial blood water that has been delivered to CSF. (**b**) Example map of estimated delivery of arterial blood–water to CSF in the human brain, capturing rapid and brain-wide blood-to-CSF water exchange.^45^

The choroid plexus (CP) or blood–cerebrospinal fluid barrier (BCSFB) is thought to be the primary source of CSF secretion in the brain and therefore represents a critical site of interest underlying brain fluid dynamics. Arterial spin labeling (ASL) MRI uses arterial blood–water as an endogenous contrast agent to image perfusion noninvasively. ASL measurement of CP perfusion may represent a valuable biomarker of CP function. Recent studies have applied standard ASL techniques to estimate CP perfusion, showing sensitivity to aging, angiogenesis, and sickle cell disease.^76,77^ An alternative approach used ASL methods combined with a long echo-time (TE) readout to quantify rates of arterial blood–water delivery to ventricular CSF across the BCSFB^78,79^ (Fig. 6a). This method was translated and expanded upon in the human brain by Petitclerc et al.^45^ This noninvasive imaging technique captured the rapid and brain-wide exchange of water that occurs between blood and CSF compartments, not just at the BCSFB in the ventricles, but also in the SAS (Fig. 6b).

#### FMRI TO MEASURE CSF FLOW.

**FIGURE 7:**
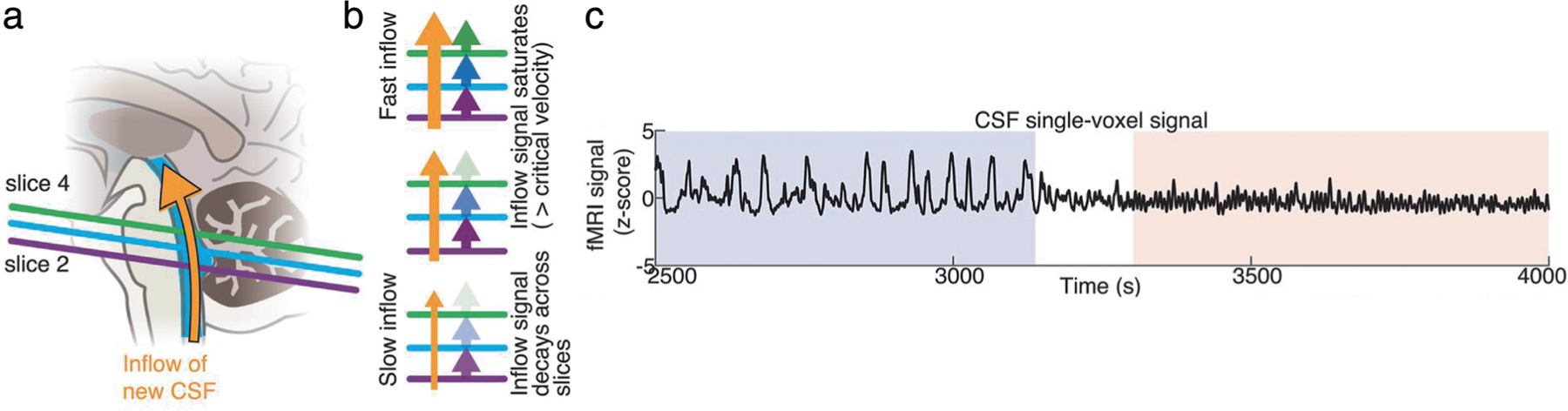
Measuring CSF dynamics with inflow effects in 2D fast fMRI. (**a**) Positioning the bottom slice of the acquisition volume of a rapid fMRI sequence to intersect the fourth ventricle leads to fresh inflow effects, with upward flowing fluid generating bright signal. (**b**) The speed of fluid flow leads to different signal intensity profiles along the bottom slices of the fMRI volume based upon the number of excitation pulses experienced by the flowing CSF before reaching a certain slice. With slow flow, the CSF will experience more RF-pulses while passing through the lowest slice(s), resulting in rapid loss of signal intensity and thus of the fresh inflow effect. With fast flow, the fresh inflow effect can be identified over multiple slices. (**c**) This method has been used to identify high-velocity pulsatile CSF flow within the fourth ventricle that specifically appears during NREM sleep. Figures adapted from Fultz et al.^47^

A key technical challenge in imaging CSF flow is that it changes rapidly over time, and many imaging techniques do not have sufficient temporal resolution to capture these dynamics. Furthermore, flow of CSF is relatively slow compared to blood, and standard velocity measurement methods such as phase contrast MRI have relatively poor sensitivity for slow flows.^80^ A recent methodological development was to repurpose fMRI acquisitions to measure CSF flow at high temporal resolution.^47^ Fast fMRI using short TRs (eg, <500 msec) leads to flow-related enhancement, in which flowing fluid exhibits brighter signals at the edge slices of the imaging volume (Fig. 7).

This phenomenon is due to the time-of-flight effect: new fluids flowing into the acquisition volume have not yet experienced RF pulses and therefore produce a bright signal in the edge slices. This property has long been known to cause high-intensity blood flow signals in fMRI data, and this effect is particularly strong when imaging at rapid timescale.^81^ While these flow signals have typically been considered to be an artifact in fMRI, it is also possible to exploit them to measure CSF flow related signal changes, by positioning the edge slices of the acquisition volume to intersect with a ventricle.^47^ These flow signals are strongest when using short TRs; however, it is also possible to use this approach to detect flow-like CSF signals in more conventional acquisitions.^82^ An additional benefit of this approach is the ability to simultaneously measure hemodynamics in the cortex with the blood oxygenation level dependent (BOLD) fMRI signal, allowing investigation of how CSF flow is coupled to vascular function throughout the brain. An important application for MRI of CSF flow is to understand the effects of sleep, as sleep is linked to higher rates of waste clearance from the brain, suggesting it may have a critical role in changing the fluid dynamics of the brain^53^(Fig. 7). These measurements have demonstrated that sleep transforms the flow of CSF in the human brain and highlight how MR-based techniques for CSF measurement can discover key properties of fluid flow in the brain.

### Contrast Agent Based Techniques

#### INTRATHECAL CONTRAST ENHANCED MRI.

**FIGURE 8:**
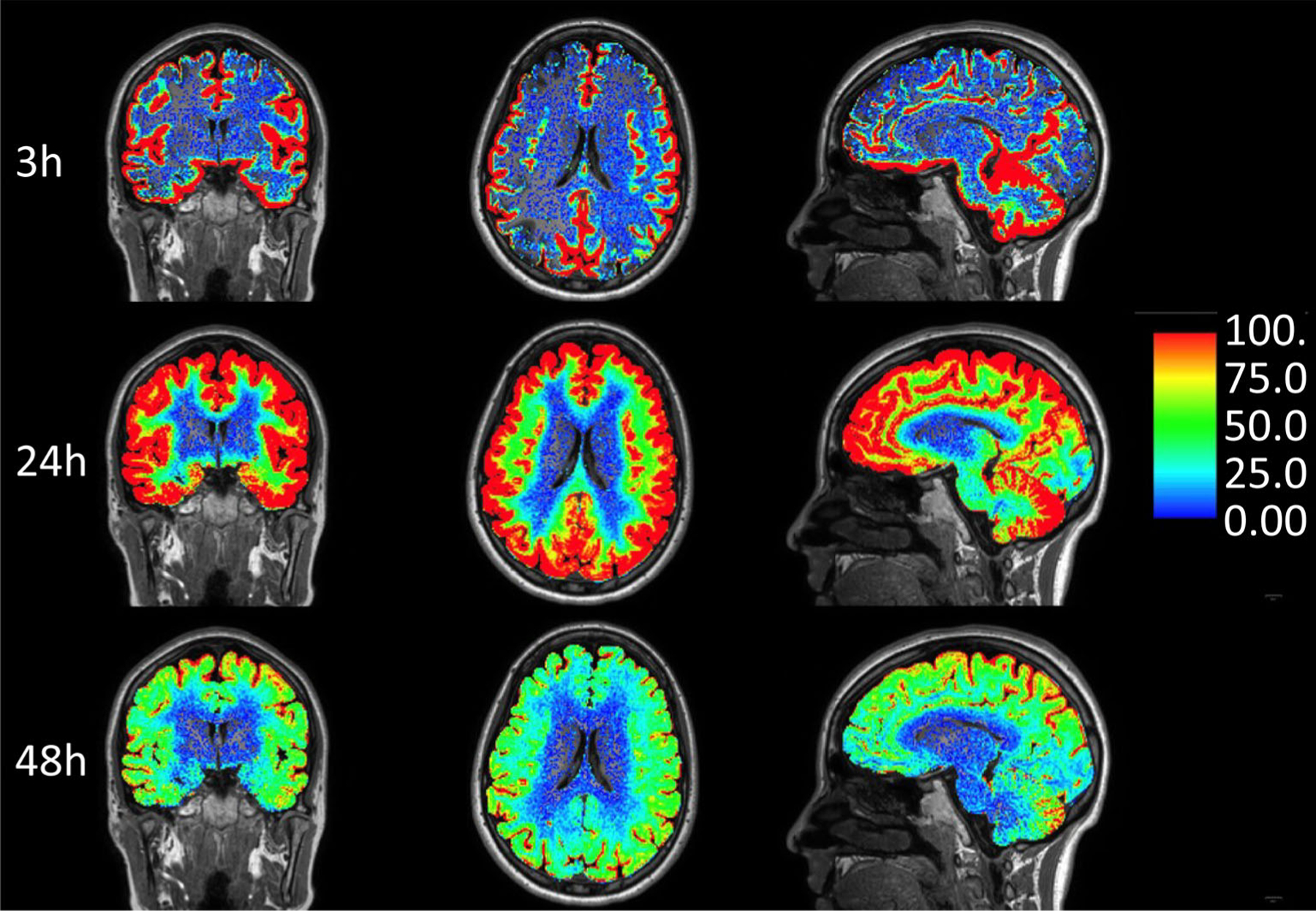
Intrathecal enhanced MRI (gMRI): Normalized, percentage T1 signal increase in the brain extravascular compartment at 3, 24, and 48 hours after administration of the MRI contrast agent gadobutrol (0.5 mmol, molecular size = 0.6 kD) in CSF at the lumbar level. Tracer enrichment in the subarachnoid and ventricular compartment is subtracted from the images. The methodology can demonstrate the potential for reaching the extra-BBB compartment in brain tissue with therapeutic drugs intrathecally, and be used to assess brain clearance rate of molecules larger than water. Tracer studies in humans have indicated that clearance from CSF is a limiting step for brain clearance. CSF tracer enrichment in brain tissue is highly dependent on tracer reaching each region of the brain surface, which can vary substantially. In this patient, who was under work-up of a pineal cyst, brain-wide enhancement in the cerebral cortex and subcortical WM occurred, but not in deep WM, which is typical. It therefore seems likely that ISF of deep cerebral WM derives from other sources than CSF, and that CSF-ISF exchange has no major role in brain molecular clearance from deep structures. Of note is also the very limited CSF tracer enhancement in the basal ganglia, which differs significantly from rodent studies, and in spite of vivid tracer enhancement in visually enlarged PVS (not shown). (Image: Vegard Vinje, PhD, Simula Research Laboratory, Oslo, Norway)

Invasive techniques can allow direct tracking of fluid transport. Macrocyclic MRI contrast agent (gadobutrol) has been utilized off-label as CSF tracer in doses of 0.25 and 0.50 mmol injected into the lumbar spinal canal, preferably mixed with saline in a total amount of 1 mL.^83^ In general, neurotoxicity has not been reported with intrathecal MRI contrast agents in doses up to 1.0 mmol, and no signs of gadolinium retention in the brain has been found.^84^ Since gadobutrol is highly hydrophilic and contained outside the intact BBB after administration in CSF (intrathecally), it can be considered a surrogate marker of how endogenous brain solutes are cleared along pathways outside the blood–brain-barrier, both from the brain and the CSF. Using consecutive T1w MRI scans through 24–48 hours after tracer injection, features of tracer enrichment and clearance within the CS compartment can be monitored (Fig. 8).

Thus far, research studies have revealed interesting findings regarding the movement of tracers from the intrathecal CSF space into the BP. First, tracer-based enhancement is vivid and reaches early around brain surfaces adjacent to large artery trunks such as the medial temporal lobe. Second, intrathecal gadobutrol can access all brain tissue regions in a centripetal fashion, thereby by-passing the brain’s intact blood–brain-barrier.^85^ Third, the degree of tracer enhancement at the brain surface was unpredictable and showed substantial interindividual variations challenging the classic concept of CSF production and absorption involving the CP and Ags. For example, intrathecal studies in patients with idiopathic normal pressure hydrocephalus (iNPH), showed early and persisting enhancement in the entire ventricular compartment whereas, tracer enhancement at the upper brain convexities was sometimes completely absent throughout the entire duration of the scan.^86^ In addition, blood levels of gadobutrol peak before peak enhancement in the upper brain convexities after injection of gadobutrol in the intrathecal lumbar space, suggesting that CSF is reabsorbed through extra-Ag pathways to a substantial degree.^87^

Physiological and anatomical differences in species are significant and must be taken into account when interpreting human data. For example, intrathecal gadolinium clearance in humans occurs over a much longer time span (days rather than hours) and CSF clearance through the nasal mucosa is minor in humans.^85,88^ Similarly, while sleep deprivation severely impairs tracer clearance in rodents, the effect is more modest in humans.^89^ Rodents have true lymphatic vessels lining the dural sinuses, in humans intrathecal tracer show direct efflux of CSF to the parasagittal dura,^59,60,90^ which also harbors lymphatic vessels, rendering for cross-talk between the peripheral immune system and brain specific antigens in this location.^91^ Gadobutrol based intrathecal MRI has the potential to serve as a surrogate marker for CSF-mediated brain clearance in several neurodegenerative diseases such as AD.

#### INTRAVENOUS (IV) CONTRAST BASED MRI.

**FIGURE 9:**
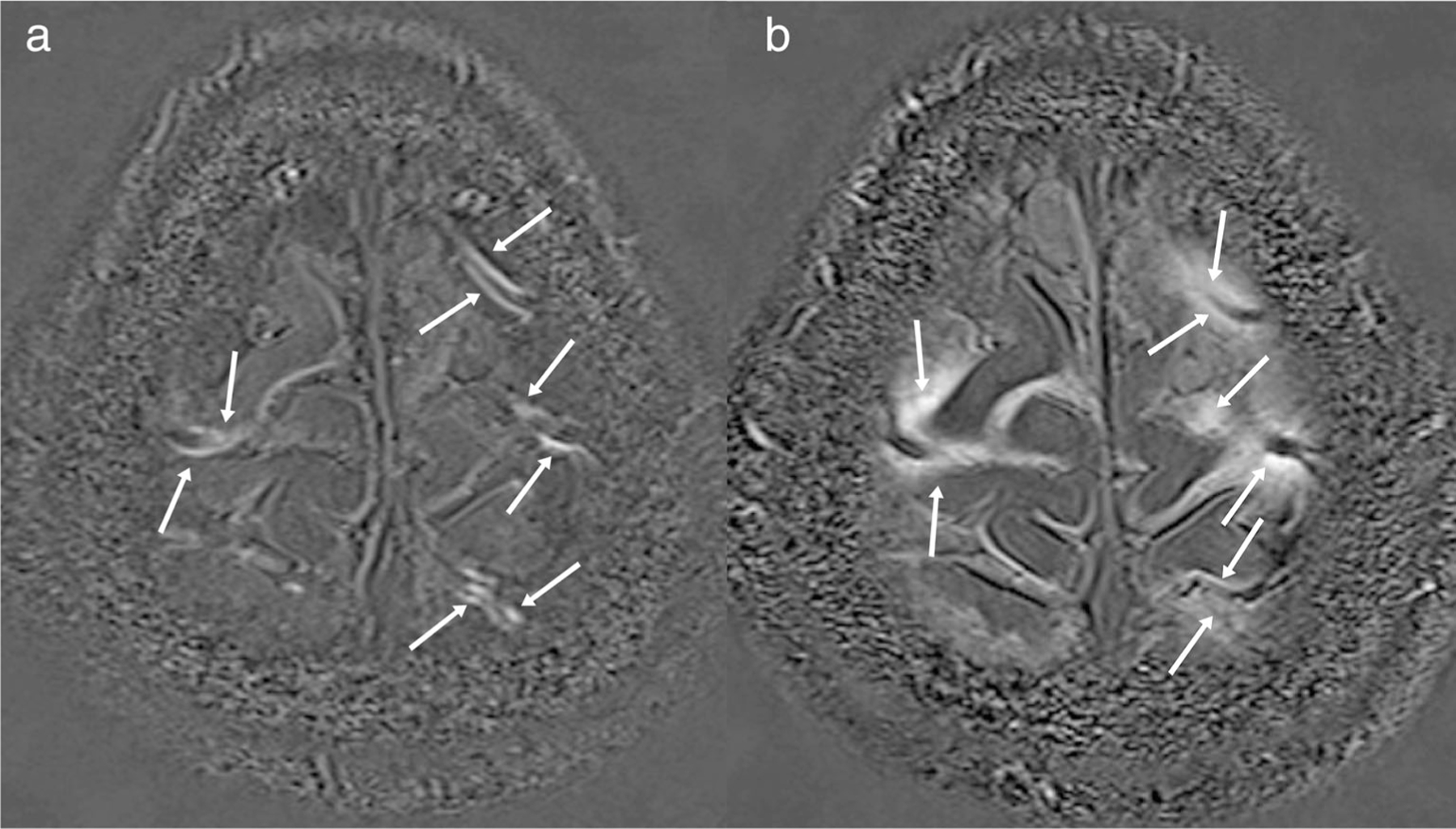
Intravenous (IV) enhanced delayed MRI: Subtracted 3D-real IR images obtained in a 60s male patient with a suspicion of endolymphatic hydrops. (**a**) Subtraction of pre-contrast 3D-real IR image from the 3D-real IR image obtained 5 minutes after IV administration of single dose gadolinium-based contrast agent (IV-GBCA). Linear structures with contrast enhancement are visualized along the cortical veins (arrows). These linear structures are speculated to be the subpial perivenous drainage routes. (**b**) Subtraction of pre-contrast 3D-real IR image from the 3D-real IR image obtained 4 hours after IV-GBCA. Contrast enhancement spread to SAS around cortical veins (arrows). The degree of the GBCA leakage to CSF has been reported to accelerate with aging.

IV administration of GBCA is less invasive and more readily achievable in clinical settings compared to intrathecal administration. A small part of IV-administered GBCA enters BP and CSF even in healthy human subjects,^92,93^ whereas BBB leakage becomes more prevalent with normal aging as well as AD.^94^ With IV-GBCA and fluid-attenuated inversion recovery (IR) images, the contrast enhancement of the inflow and outlet portions of the waste clearance system (i.e. PVS and meningeal lymphatics) can be visualized in humans.^95,96^ Subpial spaces along cortical veins have been suggested to be part of the downstream waste clearance system by IV-GBCA, and the leakage of GBCA from subpial space to SAS increases with age^97^(Fig. 9). Formation of cyst-like structures around the cortical vein near the superior sagittal sinus has been reported, although the significance is not yet completely understood.^98^ IV-GBCA based imaging has clearly shown the presence of meningeal lymphatics along superficial dural venous sinuses.^99^ It has also been suggested that the subpial space around cortical veins continues into meningeal lymphatics.^100^ A research study have shown that IV GBCA clearance increases after sleep in rodents.^101^

IV-GBCA based MRI has also been used to study clearance pathways in the eye and the ear. Delayed imaging of the eye and the ear using modified IR sequences might reveal the pathophysiology of glaucoma and Meniere’s disease.^102,103^ Linear GBCAs have been shown to be deposited in the brain by both IV and intrathecal administration. Macrocyclic GBCAs are thought to be deposited in humans in considerably smaller amounts. At least no obvious neurological effects of GBCA deposition have been demonstrated.

## Computational Modeling of Fluid Dynamics

Computational models have been successfully used to test the plausibility of hypotheses regarding neurofluid movement that cannot currently be evaluated experimentally, and to generate novel hypotheses for future experimental testing. For hypothesis testing by computational modeling, the lively discussions of recent years on the plausibility of the within-parenchyma aspect of the glymphatic system are a good example. The glymphatic hypothesis stipulates that solutes are flushed through the BP by bulk flow toward perivenous spaces, based on the tracking of fluorescent tracers by intravital two-photon fluorescent microscopy in mice.^104^ However, the driving forces underpinning tracer motion (diffusion or convection) cannot be discerned directly with this imaging modality. Computational models using diverse approaches have consistently shown that solute movement through the parenchyma via bulk flow is physically implausible, whereas experimental data is broadly consistent with diffusion-driven solute transport.^105,106^ Computational models have also identified key gaps in our understanding of the physics of CNS fluid movements and have been helpful in elucidating the conditions under which net perivascular fluid movement can occur, as well as the physical mechanisms involved. For example, numerous models have shown that while oscillatory peri-arterial bulk flow can occur, the net transport of solutes (or tracers) measured experimentally cannot be achieved by cardiac cycle-related arterial wall movements alone, and requires additional static or dynamic pressure gradients, likely in combination with diffusion (i.e., dispersion). A key challenge for the development of meaningful computational models is the need to ground them in realistic assumptions about the anatomical and mechanical parameters of the in vivo CNS, both from animal and human experiments. Overly simplified or unrealistic assumptions in models inevitably lead to unreliable conclusions, so close linkages between experimental, clinical, and modeling researchers are essential.

Modeling can also be used to complement, validate, and augment measured neurofluid flow data. For instance, while 4D PC-MRI is a powerful instrument for measuring CSF velocity in vivo,^107^ pressure gradients are the primary driving force for fluid movement around and within the central nervous system. Driving pressure gradients can be estimated from measured fluid velocities using computational fluid dynamics (CFD).^108^ Moreover, combining PC-MRI data with CFD guarantees that the *in vivo* fluid flow measurements follow the fundamental laws of physics. There are ongoing technical developments aiming to integrate key concepts from fluid mechanics into imaging workflows to improve the quality of flow measures derived from imaging, for example, by enforcing in phase contrast protocols divergence-free velocities and deep learning approaches using CFD to denoise 4D data.^109,110^

## Current Clinical Applications

### Applications in the Brain

**FIGURE 10:**
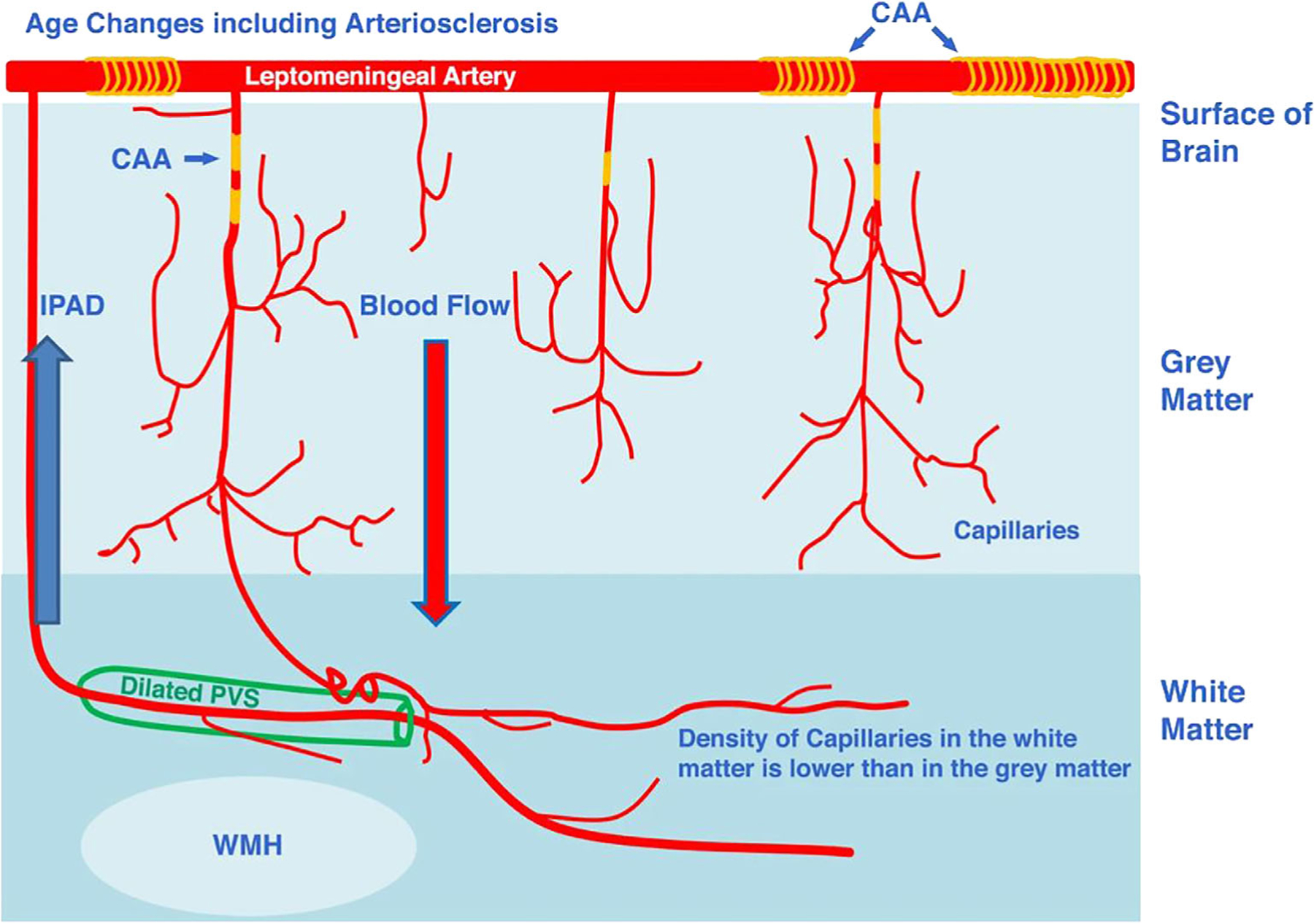
A schematic diagram representing a suggested relationship between WMH and enlarged perivascular spaces (EPVS). Note that EPVS are shown in the WM where arterioles are surrounded by a double layer of leptomeninges, facilitating the accumulation of waste products more readily in the WM rather than in the GM. CAA = cerebral amyloid angiopathy. IPAD = intramural periarterial drainage pathway (adapted from MacGregor Sharp et al^122^).

The past decade has witnessed great interest in identifying brain waste clearance pathways and their role in neurodegeneration. Two major clinically relevant MRI biomarkers are EPVS and WMH.^111,112^ Patients with CAA and AD are likely to have an increased number of EPVS in the centrum semiovale, whereas those with hypertensive SVD and cerebral hemorrhage present EPVS in the basal ganglia.^113–115^ EPVS in such patients are likely due to deposition of Aβ within the BMs of arterial walls (IPAD hypothesis) or in the extracellular interstitial space (glymphatic hypothesis)^116^ (Fig. 10) SVD and cerebral hemorrhage appear to be related to an increase in the EPVS in the basal ganglia, likely due to increased arterial stiffness, damage to blood–brain barrier and altered homeostasis leading to an increase in tissue ISF.^31^ WMH seen on T2-weighted MRI are dynamic and represent several different etiologies, one of them being increased ISF. Specific imaging such as T1 mapping and DTI analysis may further help in identifying the nature of WMH.^111^

Imaging of altered brain waste clearance pathways in humans is largely limited by the lack of spatial and temporal resolution in MRI. There appears to be some promise with GBCA based imaging over time identifying areas of slow clearance such as in patients with iNPH, endolymphatic hydrops, and glaucoma. There is general interest in applying DTI-ALPS in patients with different neurologic diseases, however, caution in the interpretation is needed.^71^ Pre- and post-GBCA T2w FLAIR imaging is gaining interest in the study of meningeal lymphatics and possible drainage pathways and has the potential to be of clinical relevance.^95,117^

**FIGURE 11:**
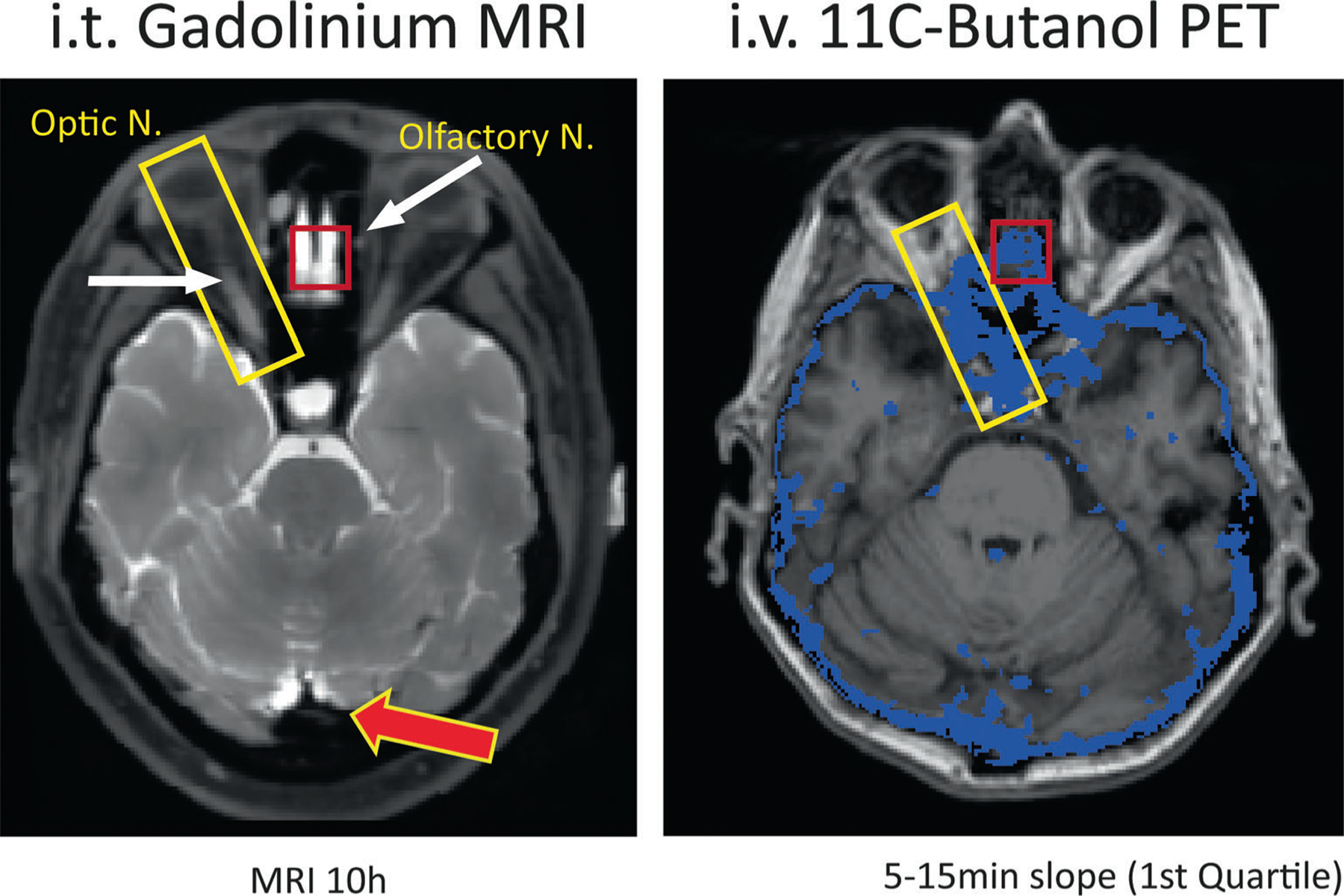
The figure demonstrates the anatomical correspondence between CSF pathways identified with the MRI contrast agent Gadolinium (left) and with the 11C-Butanol PET tracer (right). Several enhanced areas as identified by MRI and PET are highlighted in the figure, including the optic nerve and the cribriform plate at the skull base where the olfactory nerves pass. The MR image on the left was acquired 10 hours after intrathecal (lumbar) injection of 0.5 mL Magnevist (Gd-DTPA) on a 3 T GE MR750 and is a fat suppressed T1 image with a 1 × 1 × 1 mm isotropic resolution, repetition time = 602 ms, echo time = 13.3 msec, and 25.6 cm field of view. The MR-PET image on the right is derived from the IV administration of 11C-Butanol imaged dynamically (list mode) for 60 minutes with a PET SIEMENS Biograph 64, and coregistered with T1-weighted MPRAGE data from a 3 T SIEMENS Prisma. For the entire brain, the voxel-wise distribution of butanol slopes derived between 5 and 15 minutes after injection was computed. The blue area in the right image identifies the “slowest quartile” of clearing voxels superimposed on the corresponding axial MR image. The MRI image is courtesy of Drs. Jonathan Dyke Weill Cornell Medicine and Joseph Levi Chazen Hospital for Special Surgery New York.

In addition to MRI, positron emission tomography (PET) based mapping of CSF dynamics in aging and in AD was also discussed. PET studies demonstrate that 70%–85% of IV administered brain permeable radiotracers pass from the arterial blood to the interstitium and are cleared from the brain within 90 minutes.^118^ Only recently was it observed using PET that CSF egress along previously identified CSF and venous pathways could be imaged.^118^ Radiotracers with minimal specific or nonspecific brain binding sites are most interesting. In a series of studies employing limited binding PET tracers, a CSF clearance metric was defined by the decreasing ventricular concentrations of the tracer, sampled at short 10 sec intervals. The results show reduced radiotracer clearance from the lateral ventricle in association with clinically determined mild cognitive impairment and AD and in a test of the amyloid hypothesis, it was observed that ventricular CSF clearance was inversely associated with increased levels of amyloid lesions.^118,119^ The anatomical pathways for CSF drainage, as defined by PET tracer time activity curves, largely concurs with findings from gold standard intrathecal administered gadolinium contrast MRI studies. PET egress pathways have been identified for the superior sagittal sinus, optic nerve, and olfactory nerve passage via cribriform late to nasal turbinates (see Fig. 11). Such observations, if replicated and validated, suggesting an intriguing possibility of collecting CSF clearance data from available PET tracers as well as CSF sampling from a nasal swab, thus potentially avoiding lumbar puncture for brain derived proteins. PET based methods should still be considered new and do require validation and additional modeling. At present, a novel radiotracer [11C]-Butanol, that does not bind in the brain, is being tested as a potential biomarker for CSF dynamics (unpublished).

### Applications in the Spinal Cord

Spinal CSF anatomy and physiology is now understood to be much more complex and diverges from the traditional view of CSF spine physiology.^120^ Additional CSF production likely occurs within the cord tissue and vasculature and CSF absorption also occurs via arachnoid villi on nerve roots and lymphatics. The spinal SAS is characterized by abundant arachnoid septa, which can sometimes obstruct the normal flow of CSF. Flow in the SAS is influenced by cardiac pulsations, but even more so by respiratory excursions.^120^ CSF flows into the spinal cord largely driven by respiratory pressures, while fluid outflow from the cord is influenced by cardiac pulsations. Obstruction of the SAS by arachnoiditis or compression increases fluid flow into the cord.

**FIGURE 12:**
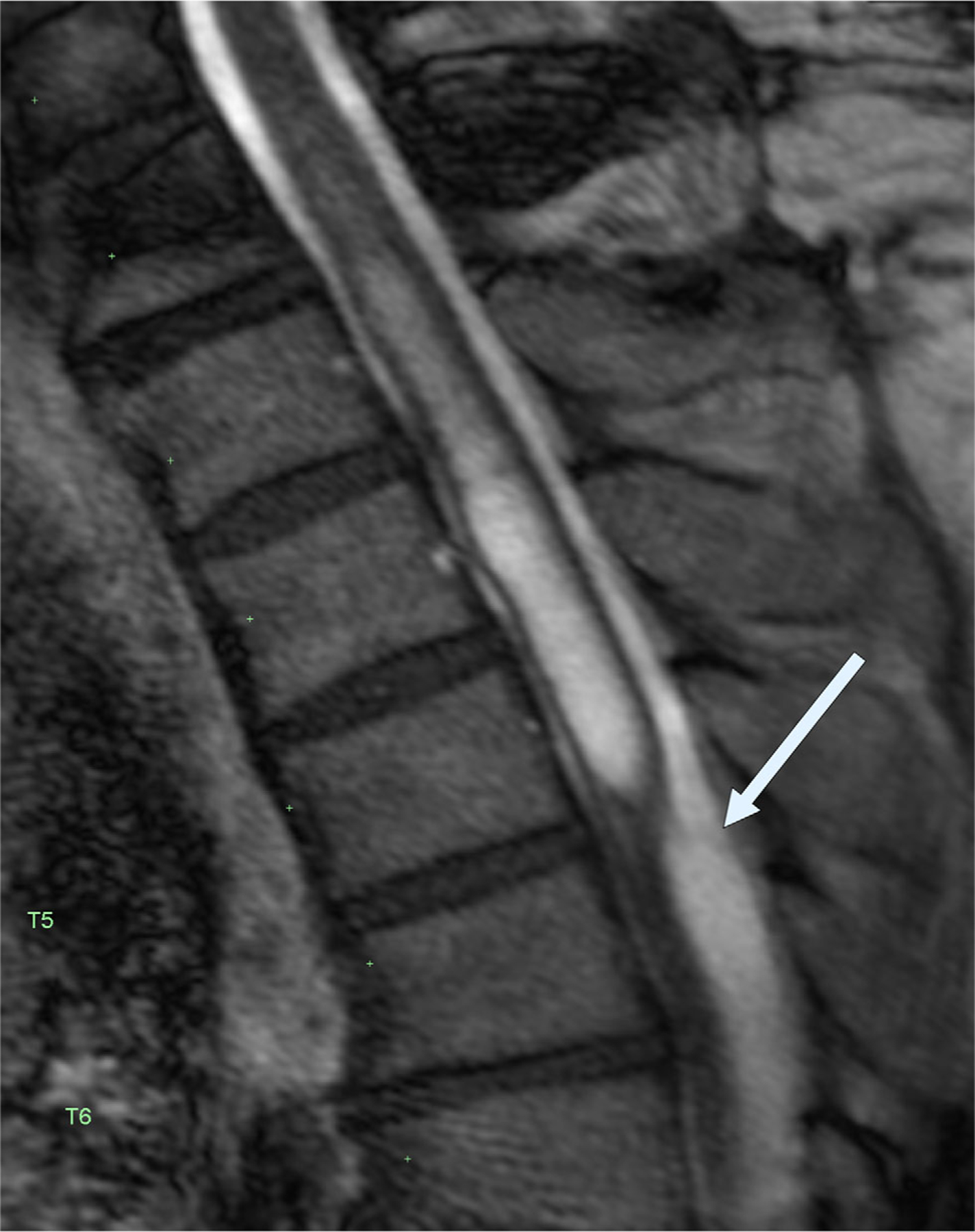
Still image from cardiac-gated, heavily T2-weighted sagittal spine MRI demonstrating an arachnoid membrane (arrow) that was the cause of a spinal cord syrinx. The membrane was not detectable on standard T2 MRI.

Pathological examples of spinal CSF physiology are Chiari malformation, syringomyelia, arachnoid cysts, and spontaneous spinal CSF leaks. The pathological mechanisms underpinning these disorders are not well understood. Syringomyelia is an accumulation of CSF in the spinal cord, yet how these cysts form remains a mystery. Dynamic imaging is proving very useful clinically, and in conjunction with computational modeling, is contributing to the understanding of this condition.^121^ Imaging CSF in the spine is difficult, with standard imaging techniques unable to demonstrate arachnoid membranes and movement of tissue and fluid. Cardiac-gated, heavily T2-weighted scans have shed light on brain and spinal cord tissue motion and are extremely useful for demonstrating abnormal arachnoid membranes that are sometimes the cause of syringomyelia (Fig. 12).

## Summary

During 2022, Neurofluids International Society of Magnetic Resonance in Medicine (ISMRM) workshop international experts with different professional backgrounds provided an overview of the current understanding of the anatomy, physiology, and imaging of the dynamics of neurofluids. Cerebral vessels not only provide our brains with the basic nutrients such as oxygen and glucose to guarantee optimal functioning of the neuroglial unit, but are also essential in removing waste generated by neuronal activity. CSF appears to *circulate* within the brain and spinal cord, thereby providing a fluidic medium for the dispersion of nutrients, mechanical protection, and removal of waste. Some CSF “escapes” via extra-parenchymal routes such as the cribriform plate and the sheaths surrounding cranial nerves. In the BP, CSF intermingles with ISF. The dynamics of all neurofluids are dependent on each other and are synchronized in a complex fashion that can be affected by several physiological processes such as sleep, respiration, cardiac activity, body posture, brain and spine compliance, and intrinsic contractile activity of the vascular smooth muscle cells (vasomotion). Several factors can act to disrupt these processes and ultimately cause neurological disorders. The role of meningeal lymphatics and other CSF exit pathways further contribute to the complexity of brain clearance pathways. In vivo MRI techniques are being actively developed to identify markers of altered clearance pathways. However, there are several blind spots in both our knowledge about brain clearance as well as in the MRI-techniques to monitor neurofluid functioning.

EPVSs are probably one of the most important indirect markers of obstructed drainage of ISF and toxins available on clinical MRI. Anatomical studies did show a link between EPVS and amyloid deposition in the cortex, further strengthening the clinical importance of EPVS.^32^ However, it remains unclear whether EPVS is truly and always pathologic.

From an imaging perspective, one of the biggest challenges for MRI remains its limited temporal and spatial resolution as well as nonspecificity of its signal. IV gadolinium-based perfusion studies allow the best temporal resolution for monitoring the inflow of tracers; however, they lack the spatial resolution required to clearly evaluate in what compartment the contrast agent resides. This raises questions like “How much of the signal change is due to intravascular enhancement?” and “What percentage is due to tracer entering tissue or CSF/ISF compartments?” Intrathecal injections show clearly the distribution of the contrast agent within the CSF-spaces and brain tissue, but suffer from a long temporal footprint (24–48 hours of intermittent observations). The combined effects of inflow and efflux of contrast agent into the cerebrum, makes it also difficult to clearly identify CSF exit pathways from the BP, even from intrathecal injections. Noninvasive imaging techniques can provide high temporal and spatial resolutions, but are only imaging the solvent of brain clearance, i.e. water in CSF and ISF, hence lacking the sensitivity and specificity for directly tracking waste products like amyloid. Moreover, residual parenchymal signal artifacts are often generated when trying to isolate signal from CSF or ISF. This can confound measurements and lead to over-interpretation of results or lack of significance.

Intrathecal use of MRI contrast agent as CSF tracer is currently used only in research, and a search for noninvasive alternatives is therefore mandated. It has been shown that CSF-to-blood clearance of gadobutrol is reduced in iNPH dementia, although with substantial variance between individuals. This measure is expected to mainly refer to the CSF-dependent meningeal lymphatic clearance capacity, which seems particularly important for clearance of tau. In future applications, intrathecal enhanced MRI as gold-standard can be compared directly with MRI sequences that may serve as noninvasive alternatives for measurement of brain clearance. Here, DTI-ALPS becomes a popular, albeit heavily debated, technique by differentiating water movement along PVSs in WM from water mobility in axonal fibers running perpendicular to these. However, it remains to be established whether movements of water in WM is a valid surrogate marker for clearance of much larger solutes such as AB and tau from the brain cortex. Moreover, the origin of DTI-ALPS signal is also a reason for careful interpretation. More recently, modified versions of well-established MRI techniques such as ASL, fMRI, and PC-MRI, are being pursued and hold promise for human neurofluid imaging.

It was clear from the workshop that after a decade of brain clearance research in rodent models, the field is fast moving toward finding imaging modalities to study brain clearance imaging in humans. Many MRI techniques originally designed for purposes other than brain clearance imaging are finding a new role in this field, further demonstrating the versatility of MRI, as an essential tool to understand our complex physiology. Finally, it emerged that there is still a lack of consensus among authors of this article, over some concepts on brain clearance imaging and the etiology of EPVS, which further demonstrates the need for concerted multidisciplinary efforts to make meaningful conclusions.

## Supplementary Material

Supplementary Video

## Figures and Tables

**TABLE 1.** Different MRI Techniques to Image Neurofluids.

Morphology/macroscopic anatomy	Conventional MRI volumetric imaging/tissue segmentation
Characterization of tissues	Relaxometry (T1/T2/T2*/T2’), magnetic resonance fingerprinting, synthetic MR
Microstructure	DTI
Perfusion/flow/BBB	DSC-MRI, DCE-MRI, and ASL MRI
CSF flow	3D, 4D phase contrast flow imaging, flow-related enhancement fMRI, intrathecal enhanced MRI
Venous blood flow	SWI and phase contrast
Brain function	BOLD fMRI
Neurometabolites/neuromodulators	Magnetic resonance spectroscopy (MRS)
Tissue compliance	MR elastography (MRE)

Abbreviations: DTI = diffusion tensor imaging; MRF = magnetic resonance fingerprinting; DSC = dynamic susceptibility contrast; DCE = dynamic contrast enhancedt; ASL = arterial spin labeling; SWI = susceptibility weighted imaging; fMRI = functional magnetic resonance imaging; MRS = magnetic resonance spectroscopy.
